# Identification and Characterization of *Campylobacter* Species in Livestock, Humans, and Water in Livestock Owning Households of Peri-urban Addis Ababa, Ethiopia: A One Health Approach

**DOI:** 10.3389/fpubh.2021.750551

**Published:** 2021-12-02

**Authors:** Gemechu Chala, Tadesse Eguale, Fufa Abunna, Daniel Asrat, Andrew Stringer

**Affiliations:** ^1^College of Veterinary Medicine, Hawassa University, Hawassa, Ethiopia; ^2^Aklilu Lemma Institute of Pathobiology, Addis Ababa University, Addis Ababa, Ethiopia; ^3^College of Veterinary Medicine and Agriculture, Addis Ababa University, Addis Ababa, Ethiopia; ^4^College of Health Sciences, Addis Ababa University, Addis Ababa, Ethiopia; ^5^College of Veterinary Medicine, North Carolina State University, Raleigh, NC, United States

**Keywords:** Ethiopia, antimicrobial resistance, *Campylobacter*, livestock, one health, zoonosis

## Abstract

*Campylobacter* is the most common cause of bacterial infectious diarrhea and acute gastroenteritis globally, and is recognized as a significant zoonotic pathogen. Antimicrobial resistance amongst *Campylobacter* isolates is a significant global concern. A cross-sectional study was conducted to identify and characterize *Campylobacter* species in humans, animals and water sources in livestock owning households of peri-urban Addis Ababa, Ethiopia; and to characterize antimicrobial resistance. A total of 519 fecal samples from humans (*n* = 99), livestock (*n* = 179), poultry (*n* = 69), and water (*n* = 172) were collected. Samples were cultured for viable *Campylobacter* spp. and multiplex PCR utilized for the identification and confirmation. Antimicrobial susceptibility of the isolates was assessed using the Kirby-Bauer disc diffusion method. *Campylobacter* spp. was detected in 67/519 (13.0%) of the total tested samples, and the household level prevalence of *Campylobacter* was 42.4%. The prevalence of *Campylobacter* spp. was: humans (10.1%), cattle (18.5%), poultry (13.0%), sheep (13.3%), goats (7.1%), and water (10.5%). *Campylobacter jejuni* and *C. fetus* were the most frequently isolated species, followed by *C. coli*. The majority of isolates obtained from human samples had co-occurrence with isolates from cattle, poultry or water samples from the same household. The use of stored water, the practice of indoor and outdoor manure collecting, and animal species *Campylobacter* positivity were significantly associated with greater odds of human *Campylobacter* spp. positivity. All *Campylobacter* isolates from humans, poultry, sheep, goats and water, and 96.0% of isolates from cattle were resistant to at least one or more of the tested antimicrobials, with 95.5% of isolates resistant to three or more classes of antimicrobials. A One Health approach is recommended to further investigate *Campylobacter* species infections, and other zoonotic infectious diseases, in the livestock owning populations in Ethiopia, where there is close interaction between humans, animals and the environment.

## Introduction

Diarrheal diseases are the leading cause of childhood illness and death in developing countries (1). Enteric pathogens including rotavirus, norovirus, *Campylobacter, Salmonella, E. coli* and *Shigella* species are known causative agents (2, 3). *Campylobacter* species are the leading zoonotic bacterial causes of human gastroenteritis and foodborne disease globally (1, 2, 4, 5). The genus *Campylobacter*, comprises of 31 species and 13 subspecies (6); of these, thermophilic *Campylobacter*, especially *C. jejuni* and *C. coli*, are the primary causes of human bacterial gastroenteritis worldwide (2, 7).

Epidemiologically, human campylobacteriosis differs across high and low-income countries. In high-income countries, symptomatic infection occurs in all age groups, whereas in low-income countries, clinical disease mostly affects children under 5 years of age, with adults rarely suffering from the disease, predominately having asymptomatic excretion (8, 9). Most *Campylobacter* species have been isolated from animals, and direct and indirect contact with animals is a known risk factor for *Campylobacter* infection, especially in children (10–12). Animals can serve both as reservoirs, and as potential sources for the contamination of food, water, and the environment (4, 13–15). Contamination of different water sources with *Campylobacter* species presents an opportunity for transmission of environment-adapted genotypes to livestock and humans (7, 13, 14, 16).

Most cases of campylobacteriosis are self-limiting, and present with enteritis, abdominal cramps, fever, nausea, and vomiting (3, 9). However, some cases have been linked to more serious complications including Guillain–Barré syndrome, Reiter's syndrome, bacteremia, and abortion (9). In severe cases of human campylobacteriosis, macrolides, and fluoroquinolones (particularly erythromycin and ciprofloxacin), and intravenous aminoglycosides are the first line antimicrobials of choice (14, 17). There is increasing antimicrobial resistance amongst *Campylobacter* isolates from various sources (18–20). This situation is more common in developing countries, where there is widespread and largely uncontrolled use of antimicrobials (11, 12, 21).

Studies on the prevalence of *Campylobacter* in Ethiopia have identified a range of 13.8–50.0% in humans, and 10.6–56.5% in animals (12, 13, 22–27). In addition, studies conducted on the antimicrobial susceptibility profiles of *Campylobacter* species, in both humans and animals in Ethiopia, have identified increased multidrug resistance of *Campylobacter* species (4, 25, 28). Most of these studies were conducted in only humans or animals, and utilized only a culture method approach for *Campylobacter* species identification and characterization. The role of different environments in Ethiopia, including water, as potential risk factors and sources for transmission of *Campylobacter* infection in humans and animals is far less known. In addition, there is limited literature regarding risk factors associated to human infection with *Campylobacter* species in Ethiopia. The identification and characterization of *Campylobacter* species in humans, animals, and water sources, is important in efforts to reduce the risk of human exposure to *Campylobacter*. The objectives of this study were the identification and characterization of *Campylobacter* spp. in humans, animals and water sources in livestock owning households of peri-urban Addis Ababa, Ethiopia; and the characterization of antimicrobial resistant isolates.

## Materials and Methods

### Study Design and Location

A cross-sectional study was conducted between December 2018 and January 2020 in Addis Ababa, Ethiopia. Addis Ababa has 10 sub-cities, of which five are considered as peri-urban areas. Akaki Kality sub-city was selected as it is characterized by a high level of livestock production, and studies have documented a high prevalence of zoonotic enteric infections (29–31). Within Akaki Kality sub-city, two woredas (Woreda 2 and 3) were purposively selected.

### Study Population

Study participants were selected from available lists of livestock-owning households in both woredas using simple random selection. Randomization of selected household lists and households was conducted using random numbers generated in a software program (Microsoft Excel 2019, Microsoft Cooperation, USA). Sample sizes were calculated for two independent populations based on an expected *Campylobacter* species prevalence of 50%, 5% margin of error, and 95% confidence level, using the total number of available livestock-owning households (227 households) in Woreda 2 and 3. The calculated sample size was 143 households and 372 animals. Households were included in the study if they owned at least one free-roaming chicken, or at least one ruminant animal. Humans and animals that were on antimicrobial treatment, or who were treated during the previous 2 weeks at the time of contact, were excluded from the study.

Of the 227 households contacted, 99 consented to participate. Households refused to participate for the following reasons: recently lost, or sold livestock (*n* = 94), no reason provided (*n* = 21), and refused to provide human or animal fecal samples (*n* = 13). Therefore, 99 households participated, providing 99 household pooled human fecal samples, and 248 household pooled livestock fecal samples. The sample size for water samples was conveniently determined based on the available water sources that was in use for both household daily activities, and drinking water for humans and animals in the study location (14).

Questionnaire data on potential risk factors associated with human infection with *Campylobacter* species were collected. Both closed and open-ended questions were utilized and translated into local languages (Afaan Oromoo and Amharic). The questionnaire was pretested on 20 households from a similar Woreda (8) in Yeka sub-city.

### Sample Collection

A total of 347 household pooled fecal samples were collected from households: humans (*n* = 99), cattle (*n* = 135), sheep (*n* = 30), goats (*n* = 14), and poultry (*n* = 69). Pooled human stool samples were collected only from individuals who were most closely associated with the management of the livestock. Water samples (*n* = 172) were collected from the 99 households: surface water (*n* = 9), municipal pipe water (*n* = 84), ground water (*n* = 16), and stored water (*n* = 63).

A sterile cotton swab moistened with nutrient broth was used to transfer ~7–10 g of the pooled fecal samples into a 15 ml screw-capped falcon tube containing Cary-Blair Transport Medium (Oxoid). Fecal samples from individual animals were collected either directly from rectum or floor of the livestock housing immediately after an animal defecated. Samples were transported to the Microbiology Laboratory of Akililu Lemma Institute of Pathobiology (ALIPB), Addis Ababa University (AAU), and processed within 4–6 h of collection.

### Isolation and Identification of *Campylobacter* Species

A selective enrichment was initially performed on all samples as previously described (32). Water samples were filtered through a 0.45 μm sterile nitrocellulose filter (Pall Corporation, Ann Arbor, MI). Membrane filtered water and homogenized human and animal fecal specimens were inoculated in 30 ml of Bolton enrichment broth containing *Campylobacter* growth supplement (HiMedia Laboratories; Mumbai, India), with 5% (v/v) defibrinated cattle blood. Tubes were then incubated for 48 h under microaerophilic condition (CO_2_, 10%; O_2_, 5%; N_2_, 85%) using Campy Gene kits (Thermo Scientific, Waltham, MA, USA) as described by Szczepanska et al. (14).

After 48 h of incubation, a loopful of culture from each sample type was spread onto plates containing modified charcoal cefoperazone deoxycholate agar (mCCDA, CM0739-Preston) and *Campylobacter* selective supplements (SR0174), and incubated under microaerophilic conditions as described earlier for 48–72 h at 42°C. After 48–72 h of incubation, all presumptive *Campylobacter* colonies were selected from each plate and checked for motility, oxidase, catalase and Gram character as previously described (7). All suspected colonies were subsequently sub-cultured onto Columbia blood agar (Difco, USA) with 5% (v/v) defibrinated cattle blood and incubated under microaerophilic condition at 42°C for 48 h. Isolates with typical colony characteristics of *Campylobacter* were then subjected to PCR analysis for confirmation and identification of *Campylobacter* species. Reference strains of *Campylobacter jejuni* and *coli* were obtained from the Ethiopian Public Health Institute and used as positive controls.

### Identification of *Campylobacter* by PCR

Genomic DNA was extracted by boiling fresh *Campylobacter* cultures grown on Columbia blood agar as previously described (33). A loopful of bacterial growth from plates were suspended in 100 μl of sterilized RNase/DNase- free water, boiled at 95°C for 15 min and cooled at 4°C until used. Genus level confirmation and identification of *Campylobacter* was conducted using multiplex-PCR (mPCR) targeting genus level and species-specific regions of the 16S rRNA, *j0414, glyA, cstA*, and *ask* gene sequences, as described previously and in Supplementary Table 1 (34). *Campylobacter* identification was conducted and isolates that were positive for the genus-specific PCR but negative for the *C. lari, C. fetus, C. coli*, and *C. jejuni*- using mPCR assay were designated as unidentified thermophilic *Campylobacter* species. In this study, a household was considered positive for *Campylobacter*, if at least one isolate was confirmed by PCR from the pooled samples of any of the animal, human, or water samples obtained.

### Antimicrobial Susceptibility of *Campylobacter* Isolates

All PCR confirmed isolates were subjected to antimicrobial susceptibility testing using the standard Kirby-Bauer disc diffusion assay on Mueller-Hinton agar (Hi Media Laboratories; Mumbai, India) according to Clinical Laboratory Standards Institute (CLSI) guidelines (35). A panel of 11 antimicrobials were used from BD BBL Sensi-Discs: ciprofloxacin (CIP) (5 μg), nalidixic acid (NA) (30 μg), erythromycin (E) (15 μg), azithromycin (AZM) (15 μg), tetracycline (Te) (30 μg), gentamicin (GM) (10 μg), ampicillin (AM) (10 μg), amoxicillin-clavulanic acid (AMC) (10 μg), chloramphenicol (C) (30 μg), and trimethoprim-sulfamethoxazole (SXT) (25 μg), and amikacin (AN) (30 μg). Isolates were defined as multidrug-resistant (MDR) when resistant to three or more classes of antimicrobials (36).

### Data Analysis and Ethical Approval

Data were analyzed using SPSS v25 (SPSS Inc, Chicago, Illinois, USA). Descriptive statistics were calculated for *Campylobacter* species prevalence, antimicrobial susceptibility and household demographic data. Chi-squared and Fisher's tests were used as appropriate to compare differences in the prevalence and antimicrobial resistance profiles between *Campylobacter* species isolates, and between strains isolated from different sources. Potential risk factors associated with the occurrence of *Campylobacter* in human samples were analyzed using univariable logistic regression. The outcome measure used was a binary variable reflecting whether a household was PCR positive for *Campylobacter*. Variables were checked for collinearity, and a backward-stepwise process was used, with covariates remaining in the model if they were statistically significant (*p* < 0.05). All variables that showed an association with the outcome variable on univariable analysis (*p* < 0.2) were considered in the final multivariable logistic regression analysis. Ethical approval was obtained (DERC/18/19/10-A) from the Research Ethics Review Committee (RERC) of the Department of Microbiology, Immunology and Parasitology (DMIP), College of Health Sciences, Addis Ababa University. Permission was obtained from the Addis Ababa Bureau of Agriculture and Livestock and Addis Abba Bureau of Health. Written informed consent was obtained prior to enrolment from the head of each household for participation in this study.

## Results

### Prevalence of *Campylobacter* Species

From the 99 households, 42 (42.4%) households were positive for *Campylobacter* in at least one of the samples tested. Of the 519 samples (347 human and animal fecal samples, and 172 water samples) from these 99 households, 67 (13.0%) were positive for *Campylobacter* species. The prevalence of *Campylobacter* species across the different samples was: human, 10 (10.1%), animal, 39 (15.7%), and water, 18 (10.5%) (Table 1). The prevalence of *Campylobacter* species was highest in surface water samples (22.5%), followed by cattle feces (18.5%), and poultry cloacal swabs (13.0%); with the lowest prevalence in goat feces (7.1%) and municipal tap water (8.3%) (Table 1).

**Table 1:** *Campylobacter* prevalence and species distribution across sample types.

**Sample type**	**Number tested**	***N* (%) positive**	**Species distribution of** ***Campylobacter*** **isolates** ***N*** **(%)**				
			***C. jejuni***	***C. coli***	***C. fetus***	***C. lari***	**Other (unidentified)**
Human feces	99	10 (10.1)	5 (50.0)	1 (10.0)	0 (0.0)	0 (0.0)	4 (40.0)
Cattle feces	135	25 (18.5)	6 (24.0)	0 (0.0)	10 (40.0)	0 (0.0)	9 (36.0)
Sheep feces	30	4 (13.3)	1 (25.0)	0 (0.0)	3 (75.0)	0 (0.0)	0 (0.0)
Goat feces	14	1 (7.1)	0 (0.0)	0 (0.0)	1 (100.0)	0 (0.0)	0 (0.0)
Poultry cloacal swabs	69	9 (13.0)	2 (22.2)	2 (22.2)	1 (11.1)	0 (0.0)	4 (44.4)
Municipal tap water	84	7 (8.3)	2 (28.6)	2 (28.6)	1 (14.3)	0 (0.0)	2 (28.6)
Stored water	63	7 (11.1)	1 (14.3)	1 (14.3)	0 (0.0)	0 (0.0)	5 (71.4)
Ground water	16	2 (12.5)	0 (0.0)	0 (0.0)	1 (50.0)	0 (0.0)	1 (50.0)
Surface water	9	2 (22.5)	0 (0.0)	0 (0.0)	0 (0.0)	0 (0.0)	2 (100.0)
Total	519	67 (13.0)	17 (25.4)	6 (9.0)	17 (25.4)	0 (0.0)	27 (40.3)

The dominant *Campylobacter* species isolated in this study were *C. jejuni* (25.4%), *C. fetus* (25.4%), and *C. coli* (9.0%). Twenty-seven isolates were categorized as unidentified *Campylobacter* species (Table 1). *C. jejuni* and *C. coli* were predominately isolated from cattle and water samples, respectively. Of the 10 *Campylobacter* species isolated from human stool samples, 5 (50%) were *C. jejuni*, and 1 (10%) was *C. coli*, with the remaining 4 (40%) unidentified species. Most isolates obtained from human stool samples were *C. jejuni. C. fetus* and *C. jejuni* were the dominant species isolated from cattle feces, with *C. fetus* being the dominant species isolated from sheep feces. From the nine *Campylobacter* isolates obtained from poultry cloacal swabs, *C. jejuni* and *C. coli* were the dominant species (Table 1). Ten *Campylobacter* species isolated from human feces had co-occurrence in other samples from the same household (Table 2).

**Table 2:** Co-occurrence of similar *Campylobacter* species in human, animals, and water samples within households.

**HHc of *Campylobacter* positive human samples**	***Campylobacter* species isolated from humans**	**Species of animals and type of water samples examined in household and** ***Campylobacter*** **species isolated**					
		**Cattle**	**Sheep**	**Poultry**	**Municipal tap water**	**Stored water**	**Ground water**
07	*C. coli*	*C. fetus*	*C. jejuni*	–	*C. coli*	–	–
15	*C. jejuni*	*C. fetus*	–	–	Other	–	–
26	*C. jejuni*	*C. jejuni*	–	–	–	–	–
41	Other			Other	–	–	–
45	*C. jejuni*	*C. jejuni*	–	–	–	–	Other
55	Other	–	–	Other	–	–	–
76	Other	–	–	–	*C. coli*		
82	*C. jejuni*	*C. jejuni*	–	–	–	Other	–
94	Other	*C. fetus*	–	–	–	–	–
96	*C. jejuni*	–	–	*C. jejuni*	–	*C. jejuni*	–

*HHc, Household code*.

Univariable logistic regression identified 11 risk factors to be associated (*p* < 0.2) with human *Campylobacter* species positivity (Table 3). Multivariable logistic regression found no association between human *Campylobacter* species positivity and self-reported gastrointestinal disease symptoms (Table 4). The collecting of manure indoors and outdoors was significantly associated with human *Campylobacter* species positivity (*p* = 0.026; OR: 38.24; 95% CI: 1.54–951.97). The washing of hands with soap before and after cooking, and taking any action to protect oneself while cleaning animal houses were associated with decreased odds of human *Campylobacter* species positivity (Table 4). In addition, the odds of human *Campylobacter* species positivity were 28.87 times less in households who did not use stored water for either drinking or food preparation (*p* = 0.037; OR: 28.87; 95% CI: 1.23–679.70) (Table 4). The multivariable analysis also identified that animal species (cattle and poultry) *Campylobacter* positivity was associated with human *Campylobacter* positivity (Table 4).

**Table 3:** Univariable logistic regression analysis of potential risk factors for human *Campylobacter* species positivity.

**Potential risk factors**	**B**	***p*-value**	**OR (95% CI)**
Municipal tap water as sole household source of water	2.13	0.048	8.41 (1.02–69.21)
Stored water as common household source of water	2.22	0.007	9.19 (1.83–46.14)
Protect oneself when cleaning animal house	−3.032	0.000	0.05 (0.011–0.22)
Heard disease transmitted from animals to human	0.292	0.729	1.34 (0.26–6.98)
Slaughter domestic animals	0.96	0.377	2.61 (0.31–21.84)
Eat and/or taste raw or undercooked meat	0.46	0.524	1.59 (0.38–6.54)
Collect manure indoor and outdoor daily	3.715	0.001	41.1 (4.85–347.5)
Wash hands with soap before and after cooking	−2.98	0.000	0.05 (0.01–0.26)
Treat drinking water	−3.5	0.001	0.03 (0.004–0.253)
Owning mixed animal species	1.903	0.077	6.71 (0.81–55.22)
Occurrence of gastrointestinal disease symptoms	1.45	0.067	4.48 (0.9–22.27)
Cull sick animals for consumption	0.46	0.535	1.58 (0.37–6.69)
Animal *Campylobacter* positivity			
Cattle	1.71	0.014	5.53 (1.4– 21.6)
Poultry	1.78	0.028	5.93 (1.21– 28.96)
Water *Campylobacter* positivity	1.86	0.008	6.42 (1.61– 25.53)

*OR, odds ratio; CI, confidence interval; B, beta coefficient (this coefficient is the degree of change in the outcome variable for every 1-unit of change in the predictor variable)*.

**Table 4:** Multivariable logistic regression analysis of significantly associated explanatory variables for human *Campylobacter* species positivity.

**Explanatory variables**	**Category**	**Number of HHs observed**	**Number (%) *Campylobacter* positive HHs**	**B**	***p*-value**	**OR (95% CI)**
Indoor-outdoor manure collecting	Yes	25	9 (36.0)	3.64	0.026	38.2 (1.54–951.97)
	No	74	1 (1.4)			
Take any action to protect oneself while cleaning animal house	Yes	83	3 (3.6)	−4.965	0.019	0.007 (0.000–0.44)
	No	16	7 (43.8)			
Wash hands with soap before and after cooking	Yes	76	2 (2.6)	−4.168	0.021	0.02 (0.000–0.531)
	No	23	8 (34.8)			
Stored water as common source of water for household	Yes	35	8 (22.9)	3.363	0.037	28.87 (1.23–679.7)
	No	64	2 (3.1)			
Cattle *Campylobacter* positivity per HH	Positive	25	6 (24.0)^*^	2.019	0.022	7.53 (1.33–42.55)
	Negative	63	4 (6.35)^#^			
Poultry *Campylobacter* positivity per HH	Positive	9	3 (33.3)^*^	2.934	0.005	18.80 (2.38–148.41)
	Negative	27	7 (25.93)^#^			

*B, beta coefficient (this coefficient is the degree of change in the outcome variable for every 1-unit of change in the predictor variable); OR, Odds Ratio; CI, Confidence Interval; HH, Household*.

* *Number of Campylobacter spp. positive households when household's cattle or poultry found positive for Campylobacter spp*.

# *Number of Campylobacter spp. positive households when household's cattle or poultry found negative for Campylobacter spp*.

### Antimicrobial Susceptibility Profiles of *Campylobacter* Isolates

Over 98% of the isolates were resistant to one or more antimicrobial agents. With the exception of *Campylobacter* isolates from cattle, where 96% showed resistance to one or more tested antimicrobials, all isolates from humans, sheep, goats, poultry, and water were resistant to at least one of the tested antimicrobials (Table 5). Most isolates were resistant to amikacin (79.1%) followed by amoxicillin-clavulanic acid (70.1%), tetracycline (67.2%), ampicillin (64.2%), and Nalidixic acid (64.2%) (Table 5). A single isolate was found to be resistant to only erythromycin and amikacin, and only one isolate was resistant to only erythromycin, azithromycin and amikacin (Table 5).

**Table 5:** Antimicrobial susceptibility profiles of *Campylobacter* species isolated from various sources.

**Antimicrobial classes**	**Antimicrobial**	**Number (%) of resistant** ***Campylobacter*** **from different sources**						
		**Human (*n* = 10)**	**Cattle (*n* = 25)**	**Sheep (*n* = 4)**	**Goat (*n* = 1)**	**Poultry (*n* = 9)**	**Water (*n* = 18)**	**Total (*n* = 67)**
Aminoglycosides	Amikacin	9 (90.0)	18 (72.0)	4 (100.0)	1 (100.0)	7 (77.8)	14 (77.8)	53 (79.1)
	Gentamycin	3 (30.0)	9 (36.0)	2 (50.0)	0 (0.0)	2 (33.3)	4 (22.2)	21 (31.3)
Macrolides	Azithromycin	7 (70.0)	14 (56.0)	2 (50.0)	1 (100.0)	5 (55.6)	11 (61.1)	40 (59.7)
	Erythromycin	8 (80.0)	14 (56.0)	1 (25.0)	1 (100.0)	6 (66.7)	11 (61.1)	41 (61.2)
Penicillin	Amoxicillin-clavulanic acid	7 (70.0)	15 (60.0)	2 (50.0)	0 (0.0)	8 (88.9)	15 (83.3)	47 (70.1)
	Ampicillin	6 (60.0)	15 (60.0)	2 (50.0)	0 (0.0)	6 (66.7)	14 (77.8)	43 (64.2)
Phenicol	Chloramphenicol	2 (20.0)	5 (20.0)	2 (50.0)	1 (100.0)	0 (0.0)	3 (16.7)	13 (19.4)
Potentiated sulfonamides	Sulfamethoxazole/trimethoprim	2 (20.0)	13 (52.0)	3 (75.0)	1 (100.0)	2 (33.3)	6 (33.3)	28 (41.8)
Quinolones	Ciprofloxacin	5 (50.0)	14 (56.0)	4 (100.0)	1 (100.0)	7 (77.8)	11 (61.1)	42 (62.7)
	Nalidixic acid	6 (60.0)	13 (52.0)	1 (25.0)	1 (100.0)	6 (66.7)	16 (88.9)	43 (64.2)
Tetracycline	Tetracycline	7 (70.0)	17 (68.0)	4 (100.0)	1 (100.0)	5 (55.6)	11 (61.1)	45 (67.2)

The resistance to individual antimicrobials across *Campylobacter* species ranging from 11.1 to 100.0% (Table 6). *C. coli* exhibited wider resistance to most of the tested antimicrobials than *C. jejuni* and other isolates. There was a significant difference in the resistance of *C. jejuni* and *C. fetus* to nalidixic acid, amoxicillin-clavulanic acid, erythromycin, and ampicillin (*p* < 0.05). However, no statistically significant difference was observed for the other antimicrobials across the different *Campylobacter* species. All poultry isolates were susceptible to chloramphenicol, with one unidentified *Campylobacter* species isolate from cattle pan-susceptible, and one *C. coli* isolate from water resistant to all tested antimicrobials.

**Table 6:** Resistance of antimicrobials across *Campylobacter* species.

***Campylobacter* species (*n*)**	**Antibiotic resistant profile (%)**											
	**AM**	**AMC**	**AZM**	**AN**	**C**	**CIP**	**E**	**GM**	**NA**	**SXT**	**Te**	**MDR**
*C. jejuni* (17)	88	94	77	82	18	88	88	24	35	35	88	94
*C. coli* (6)	88	100	83	50	17	67	83	33	100	50	100	100
*C. fetus* (17)	41	53	53	82	35	59	35	29	71	59	71	100
Other Species (27)	59	60	48	82	11	48	56	37	70	33	44	7

*AM, Ampicillin; AMC, Amoxicillin-Clavulanic acid; AN, Amikacin; AZM, Azithromycin; C, Chloramphenicol; CIP, Ciprofloxacin; E, Erythromycin; GM, Gentamycin; NA, Nalidixic acid; SXT, Sulfamethoxazole; Te, Tetracycline; MDR, Multidrug resistance*.

Sixty-four (95.5%) of the isolates were found to be resistant to three or more antimicrobial classes, and were thus considered multidrug resistant. They were observed across the following sources: humans, 8 (12.5%); cattle, 24 (37.5%); sheep, 4 (6.3%); goats, 1 (1.6%); poultry, 9 (14.1%); and water, 18 (28.1%) (Table 7). Isolates from humans, cattle and poultry were resistant to 3–6 antimicrobial classes, whereas isolates from water and sheep were resistant to 3–7 and 4–7 antimicrobial classes, respectively. A single isolate from goats was resistant to six different antimicrobial classes.

**Table 7:** Antimicrobial resistance profiles of *Campylobacter* species isolated from animals, humans, and water in peri-urban Addis Ababa, Ethiopia.

***Campylobacter* species**	**Source**							**Antimicrobial resistance profile and source**
	**Cattle**	**Poultry**	**Sheep**	**Goat**	**Human**	**Water**	**Total**	
*C. jejuni*	6	2	1	–	5	3	17	AZM-AN-E (**H**), AM-AMC-AN-AZM-C-CIP-E-GM-Te (**H**), AM-AMC-AN-AZM-C-CIP-E-Te (**H**), AM-AMC-AN-CIP-NA-SXT-Te (**H**), AM-AMC-AZM-CIP-E-NA-Te (1**H**, 1**W**, 1**P**), AM-AMC-AZM-C-E-Te (**C**), AM-AMC-AN-CIP-E-GM-Te (**C**), AM-AMC-AZM-CIP-E-SXT-Te (**C**), AM-AMC-AN-AZM-CIP-E-SXT-Te (1**C**, 1**P**), AM-AMC-AN-AZM-CIP-E-GM-NA-SXT (**C**), AM-AMC-AZM-C-CIP-E-Te (**C**), AM-AMC-AN-CIP-E-Te (**W**), AM-AMC-AN-AZM-CIP-GM-NA-Te (**W**), AM-AMC-AN-CIP-E-SXT-Te (**S**)
*C. coli*	–	2	–	–	1	3	6	AM-AMC-AZM-CIP-E-NA-SXT-Te (**H**), AMC-NA-Te (**W**), AM-AMC-AN-AZM-E-NA-Te (**W**), AM-AMC-AN-AZM-C-CIP-E-GM-NA-SXT-Te (**W**)^**β**^, AM-AMC-AZM-CIP-E-NA-Te (**P**), AM-AMC-AN-AZM-CIP-E-GM-NA-SXT-Te (**P**)
*C. fetus*	10	1	3	1	–	2	17	AN-C-CIP-GM-NA-SXT-Te (**C**), AM-AMC-AN-C-CIP-E-NA-Te (**C**), AM-AN-AZM-NA-SXT (**C**), AM-AMC-AN-AZM-SXT (**C**), AM-AMC-CIP-E-GM-NA-Te (**C**), AM-AMC-AN-E-SXT (**C**), AZM-E-NA-SXT-Te (**C**), AN-NA-SXT-Te (**C**), AZM-E-NA-Te (**C**), AMC-AN-AZM-CIP-NA-Te (**C**), AM-AN-AZM-C-CIP-SXT-Te (**S**), AN-CIP-GM-NA-SXT-Te (**S**), AMC-AN-AZM-C-CIP-GM-Te (**S**), AN-AZM-CIP-E-NA-SXT-Te (**G**), AMC-AN-CIP-NA-SXT (**W**), AM-AMC-AN-AZM-CIP-GM-NA-Te (**W**), AMC-AN-CIP (**P**)
Other species	9	4	–	–	4	10	27	AN-E (**H**), AN-AZM-GM-NA (**H**), AM-AMC-AN-AZM-E-GM-NA-Te (**H**), AM-AMC-AN-C-CIP-E-NA-Te (**H**), AM-AN-AZM-C-E-NA-SXT-Te (**W**), AM-AMC-AZM-AN-CIP-E-NA (**W**), CIP-NA-Te (**W**), AN-E-GM-Te (**C**), AM-AMC-AZM-CIP-E-NA-SXT-Te (**W**), AM-AMC-AN-AZM-CIP-NA (**W**), AM-AMC-E-NA-SXT (**W**), AN-AZM-CIP-E-NA (**W**), AM-AMC-AN-CIP-E-SXT-Te (1**W**, 1**C**), AM-AMC-AN-C-CIP-NA (**W**), AM-AN-AZM-GM-NA-SXT-Te (**C**), AN-AZM-CIP-GM-NA-Te (**C**), AMC-AN-AZM-GM (**C**), AM-AMC-AN-CIP-NA-SXT (**C**), AN-AZM-CIP-E-NA-SXT-Te (**C**), AM-AMC-AN-CIP-GM (**C**), AM-AMC-CIP-Te (**P**), AN-CIP-E-NA (**P**), AMC-AN-GM-NA-SXT (**P**), AM-AMC-AN-AZM-E-GM-NA (1**P**, 1**W**), Pan susceptible (1**C**)

*AM, Ampicillin; AMC, Amoxicillin-clavulanic acid; AN, Amikacin; AZM, Azithromycin; C, Chloramphenicol; CIP, Ciprofloxacin; E, Erythromycin; GM, Gentamycin; NA, Nalidixic acid; SXT, Sulfamethoxazole + trimethoprim; Te, Tetracycline; **C**, Cattle; **S**, Sheep; **H**, Human; **P**, poultry; **W**, water; **G**, Goat; **β**, Resistant to all tested antimicrobials*.

Regardless of the source, all *C. coli* and *C. fetus* isolates demonstrated a multidrug resistance profile, whereas a multidrug resistance profile was observed in 94.1% (16/17) of *C. jejuni* and 92.6% (25/27) of unidentified *Campylobacter* species (Table 6). Fifty-four different multidrug resistance profiles, ranging from 3 to 7 different antimicrobial classes were observed (Table 7). The most common multidrug resistance profile in isolates was AM-AMC-AZM-CIP-E-NA-Te (7.4%), followed by AM-AMC-AZM-AN-CIP-E-SXT-Te (5.6%), and AM-AMC-AN-CIP-E-SXT-Te (5.6%). Resistance to macrolides (erythromycin and azithromycin), was the most dominant antimicrobial class appearing in 74.1% (37) of the fifty-four observed multidrug resistance patterns (Table 7).

## Discussion

Of the 99 households in this study, 42.4% were positive for *Campylobacter* species in at least one of the household samples tested. The prevalence of *Campylobacter* species in humans in our study, was similar to a number of previous studies in Ethiopia (20, 38), Poland (14), and Tanzania (39). In contrast, studies in Ethiopia by Terefe et al. (22), Tafa et al. (23), and Lengerh et al. (40) reported higher prevalences than our study. A higher prevalence was also reported by Chuma et al. (41) in Tanzania, and Schiaffino et al. (36) in Peru, whereas Rawat et al. (37) and Meistere et al. (42) reported a lower prevalence in Latvia and India. The variation in prevalence across the studies might be due to differences in study methodologies, periodical and environmental variations, and differences in the study groups (14, 42).

*Campylobacter* species prevalence observed in cattle in this study is consistent with a previous Ethiopian study (43). However, it is a higher prevalence than reported by Hagos et al. (4) and Kassa et al. (25) in Ethiopia, and a lower prevalence than reported by Abamecha et al. (44) in Ethiopia. Comparable results were also reported from the Republic of Korea (45) and Latvia (42). Prevalence in our study was higher than those reported from Iran (46), and Ghana, but lower than reports from Republic of Korea (47), and the USA (48). The prevalence of *Campylobacter* species observed in poultry in this study is consistent with the report from Latvia (42), but lower than previous studies in Ethiopia (12, 25), Tanzania (41), Kenya (49), and Cambodia (15). However, the prevalence in our study is higher than the reports by Pires et al. (50) from California, USA, and Rawat et al. (37) from India.

The variation in prevalence of *Campylobacter* species in animals across these studies might be explained by the differences in study methodology and duration, seasonality, animal management system, sanitary practices, and agro-ecological variations (15, 41). Certain studies, like Hagos et al. (4), reported a prevalence from meat products rather than live animals. Our study was a community level study, utilizing a pooled sampling approach, where all sampling units were asymptomatic at the time of sample collection. In contrast, many previous studies conducted in Ethiopia were based on symptomatic human subjects, and/or farm-level animal studies that potentially resulted in the observed variation in prevalence.

Our study utilized both a culture and molecular approach for the isolation and identification of *Campylobacter* species. The majority of previous studies in Ethiopia used only the culture and biochemical test approach for the isolation and identification of *Campylobacter*. This latter approach has the potential to be less sensitive and specific, and could be a reason for the observed differences in prevalence (51–53). Nevertheless, the higher prevalence of zoonotic thermophilic *Campylobacter* species observed in livestock in our study is of considerable concern, as poultry and other livestock species move freely around the household, contaminating the environment, and are a source of infection for humans, especially children.

Of all the sample types in our study, the prevalence of *Campylobacter* species was highest in surface water samples (22.5%). The overall prevalence of *Campylobacter* species observed in all water types in our study, is consistent with a study from Turkey, but lower than studies from South Africa (7) and Poland (14). The variation across studies might be attributed to differences in the sources of water, water collection approach, season, geographical location, and method of isolation. In addition, our study area is impacted by both agricultural operations (cattle, poultry, and vegetable production), and urban wastewater treatment, which may influence the prevalence of *Campylobacter* and other microorganisms (31, 54). Although the contribution of water to the burden of *Campylobacter* infection in humans might be unknown (55), the results of our study provide information regarding the potential transmission of *Campylobacter* species between animals, humans and water sources. More importantly, our study revealed a high prevalence of thermophilic *Campylobacter* species in municipal tap water, indicating a potential risk for human infection. However, this result should be interpreted with caution, as no information was obtained on whether the source water was treated. The significantly higher isolation of *Campylobacter* from households using stored water, is consistent with previous studies (7, 56). Studies have shown that untreated stored water is a significant source of *Campylobacter* infections and outbreaks (16, 55). Risk factors including the source of water and type of storage container have been linked to the poor microbial quality of stored household water (56).

In our study, *C. jejuni* was the most frequently isolated species from all sample types, except for cattle and poultry isolates. This is consistent with previous studies in Ethiopia (22, 25) and other countries, including South Africa and California, USA (7, 50). *C. jejuni* is more prevalent, and has a longer viability in the environment compared to other thermophilic *Campylobacter* species, therefore increasing its chance of recovery (33, 50). This difference could also occur due to variations in the mechanism of pathogenesis and elimination amongst the different thermophilic *Campylobacter* species within the host cells (14). Differences in the isolation of *Campylobacter* species might also be related to their actual compositional variations in local environments. *C. lari* was not recovered in our study, consistent with previous studies in Ethiopia (22, 28, 33, 50). As demonstrated in other studies (7, 12, 39), our study also highlighted that *Campylobacter* species isolated from human feces also co-occurred in other samples (predominately cattle, poultry, and water) from the same household, suggesting that cattle, water and poultry are potentially the main sources of human *Campylobacter* infection. It is also possible, that humans could have infected the cattle, poultry and stored water.

The association of indoor and outdoor manure collecting with increased odds of human *Campylobacter* species positivity is consistent with previous studies (15, 47). *Campylobacter* can survive at variable rates in stored manure, and even in composted manure [reviewed in (50, 57)]. Consequently, human exposure to *Campylobacter* species may occur when drinking or ingesting of contaminated products (47). The negative association of taking any specific action to protect oneself while cleaning an animal house, or washing hands with soap before and after cooking, with *Campylobacter* species positivity is similar to a previous study (15). Poor hygiene and sanitation are associated with increased odds of multiple adverse health outcomes (58). However, our study indicated that there was no significant association between the consumption of raw or under cooked meat, and owning different animal species with human *Campylobacter* species positivity.

Nearly all isolates in our study were resistant to one or more antimicrobials. Most isolates were resistant to amikacin (79.1%), followed by amoxicillin-clavulanic acid (70.1%), whereas a lower resistance was observed to chloramphenicol (19.4%), followed by gentamicin (31.3%). Similar results have been reported previously in Ethiopia (59) and Iran (60). The level of resistance to nalidixic acid (64.2%) and ciprofloxacin (62.7%) observed in our study are consistent with other studies in Ethiopia (44), Poland (61), and Kenya (49). Resistance to erythromycin (61.2%) and azithromycin (59.7%) observed in this study, is consistent with results found in other previous studies (33, 44, 60), but higher than other studies (14, 23, 40). In our study, resistance observed to tetracycline (67.2%), ampicillin (64.2%), and sulfamethoxazole-trimethoprim (41.8%) is consistent with previous studies (7, 40), but lower resistance has been previously reported (25, 37).

In our study, *C. coli* was highly resistant to nalidixic acid, amoxicillin-clavulanic acid, azithromycin, and ampicillin, compared to other species. Whereas, *C. jejuni* showed relatively higher resistance to erythromycin, ciprofloxacin and amikacin than other species. These differences could be due to greater use of these drugs in humans, and that humans are commonly infected with these species of *Campylobacter* (62). This is consistent with studies from Ethiopia (13), South Africa (7, 63), Tunisia (64), but differs from other studies in Ethiopia (59), Egypt (65), and England (66).

The high number of multidrug resistant *Campylobacter* isolates observed in our study is consistent with previous studies (7, 44). However, lower antimicrobial resistance has been previously reported in Ethiopia (25), Iran (46), and Tanzania (33). One potential hypothesis for the high resistance exhibited by *C. coli*, compared to other *Campylobacter* species, may be that *C. coli* strains can acquire resistance genes horizontally more effectively than other species, and chromosomally encoded target genes could mutate faster in *C. coli* (67, 68). However, we need to be mindful about our interpretation, given the low number of *C. coli* isolates in our study.

The higher antimicrobial resistance observed in our study may potentially be due to the overuse, and inappropriate use, of antimicrobial agents in both human and animals. To our knowledge, oxytetracycline and penicillin (alone or in combination with streptomycin) are used widely in livestock and poultry in Ethiopia. This use contributes to increased selection of resistant *Campylobacter* species (69, 70). Extensive use of antimicrobials in the animal industry for prophylaxis, and growth promotion, has been associated with increased resistance (14, 65, 71). Due to the close human and animal interaction in our study area, there is the potential for antimicrobial resistant strains, or resistant genetic markers originating from humans, to be transmitted to animals. The differences in the isolation of *Campylobacter* species across the different sources, may make it challenging to compare the levels of resistance between the sources. Nonetheless, observed resistance discrepancies in our study, compared to previous studies, could be due to differences in exposure rates of the bacteria to the different antimicrobials. The inevitable human-animal-environment interaction in our study, along with inappropriate use of antimicrobials in both animals and humans in Ethiopia, might potentially lead to increased selection pressures for resistant strains.

The challenges presented by infectious diseases and antimicrobial resistance are multifaceted, and it is critically important to address these issues using a One Health approach (72, 73). One Health interventions advocate close intersectoral cooperation, interdisciplinary expertise, and the involvement, and empowerment of multiple stakeholders (74, 75). Our study demonstrated that *C. jejuni*, C. *coli*, C. *fetus*, and unidentified *Campylobacter* species were prevalent in the study area. The relatively high proportion of *Campylobacter* species in both livestock and water samples are potential risks for human *Campylobacter* infection. The high prevalence of *Campylobacter* species isolated from various water sources, highlights the need for further work to identify for how long *Campylobacter* species persist in these water sources, and to ascertain the transmission of *Campylobacter* within the environment. Multi-drug resistant zoonotic *Campylobacter* species were prevalent in animals, humans and environment in the peri-urban livestock owning households of Addis Ababa, Ethiopia. Recognizing the significant implications of antimicrobial resistance, it is important for Ethiopia to develop and implement a national plan to advance the rational use of antimicrobials utilizing a One Health approach. A One Health approach is recommended to further investigate *Campylobacter* species infections, and other zoonotic infectious agents, in the livestock owning populations in Ethiopia, where there is close interaction between humans, animals and the environment.

## Data Availability Statement

The raw data supporting the conclusions of this article will be made available by the authors, without undue reservation.

## Ethics Statement

The studies involving human participants were reviewed and approved by the Research Ethics Review Committee (RERC) of the Department of Microbiology, Immunology and Parasitology (DMIP), College of Health Sciences, Addis Ababa University (DERC/18/19/10-A). Written informed consent to participate in this study was provided by the participants' legal guardian/next of kin. The animal study was reviewed and approved by the Research Ethics Review Committee (RERC) of the Department of Microbiology, Immunology and Parasitology (DMIP), College of Health Sciences, Addis Ababa University (DERC/18/19/10-A). Permission was obtained from the Addis Ababa Bureau of Agriculture and Livestock and Addis Abba Bureau of Health. Written informed consent was obtained from the owners for the participation of their animals in this study.

## Author Contributions

GC, TE, FA, DA, and AS conceived and designed the study and designed the survey. GC led the sample collection and survey implementation. GC, TE, and DA supervised the laboratory analysis. GC analyzed the data, with supervision from AS and TE. GC and AS wrote the manuscript. All authors contributed to the revision of the manuscript.

## Funding

Funding provided by North Carolina State University.

## Conflict of Interest

The authors declare that the research was conducted in the absence of any commercial or financial relationships that could be construed as a potential conflict of interest.

## Publisher's Note

All claims expressed in this article are solely those of the authors and do not necessarily represent those of their affiliated organizations, or those of the publisher, the editors and the reviewers. Any product that may be evaluated in this article, or claim that may be made by its manufacturer, is not guaranteed or endorsed by the publisher.
