# Supplementary material for: Identification and Characterization of Campylobacter Species in Livestock, Humans, and Water in Livestock Owning Households of Peri-urban Addis Ababa, Ethiopia: A One Health Approach
Source: Front Public Health. 2021 Dec 2;9:750551. doi: 10.3389/fpubh.2021.750551 (PMC8677049; doi:10.3389/fpubh.2021.750551)
Supplement: Supplementary file 1 [file Table_1.DOCX]

Supplementary Table 1: Primer pairs used for detection and speciation of Campylobacter species using multiplex PCR and expected amplicon size (Yamazaki-Matsune et al., 2007)

| Species | Expected amplicon Size (bp) | Target gene | Primer | Sequence (5’ to 3’) |
| --- | --- | --- | --- | --- |
| Genus *Campylobacter* | 816 | 16S rRNA | C412F  C1228R | 5’-GGATGACACTTTTCGGAGC-3’  5’-CATTGTAGCACGTGTGTC-3’ |
| *C. coli* | 502 | *Ask*^¥^ | CC18F  CC519R | 5’-GGTATGATTTCTACAAAGCGAG-3’  5’-ATAAAAGACTATCGTCGCGTG-3’ |
| *C. jejuni* | 161 | *cj0414*^ƒ^ | C-1  C-3 | 5’-CAAATAAAGTTAGAGGTAGAATGT-3’  5’-CCATAAGCACTAGCTAGCTGAT-3’ |
| *C. lari* | 251 | *glyA*^β^ | CLF  CLR | 5’-TAGAGAGATAGCAAAAGAGA-3’  5’-TACACATAATAATCCCACCC-3’ |
| *C. fetus* | 359 | *cstA*^⸸^ | MG3F  MG3F | 5’-GGTAGCCGCAGCTGCTAAGAT-3’  5’-AGCCAGTAACGCATATTATAGTAG-3’ |

**^¥^** presumed to encode for aspartokinase gene

**^⸸^** presumed to encodeforcarbon starvation protein A gene

**^ƒ^**presumed to encode an oxidoreductase

**^β^**presumed to encode for serine hydroxymethyltransferase
